# Mental health care in Nepal: current situation and challenges for development of a district mental health care plan

**DOI:** 10.1186/s13031-014-0030-5

**Published:** 2015-02-06

**Authors:** Nagendra P Luitel, Mark JD Jordans, Anup Adhikari, Nawaraj Upadhaya, Charlotte Hanlon, Crick Lund, Ivan H Komproe

**Affiliations:** Research Department, Transcultural Psychosocial Organization (TPO), Kathmandu, Nepal; Research and Development Department, HealthNet TPO, Lizzy Ansinghstraat 163, 1073 RG, Amsterdam, The Netherlands; Centre for Global Mental Health, Institute of Psychiatry, Psychology and Neuroscience, King’s College London, Box P029 De Crespigny Park, London, SE5 8AF UK; Department of Psychiatry, School of Medicine, College of Health Sciences, Addis Ababa University, Addis Ababa, Ethiopia; Alan J Flisher Centre for Public Mental Health, Department of Psychiatry and Mental Health, University of Cape Town, Cape Town, South Africa; Faculty of Social and Behavioural Sciences, Utrecht University, Utrecht, The Netherlands

**Keywords:** Mental health, Situation analysis, Integration of mental health into PHC, Mental health care plan, Nepal

## Abstract

**Background:**

Globally mental health problems are a serious public health concern. Currently four out of five people with severe mental illness in Low and Middle Income Countries (LMIC) receive no effective treatment. There is an urgent need to address this enormous treatment gap. Changing the focus of specialist mental health workers (psychiatrists and psychologists) from only service delivery to also designing and managing mental health services; building clinical capacity of the primary health care (PHC) workers, and providing supervision and quality assurance of mental health services may help in scaling up mental health services in LMICs. Little is known however, about the mental health policy and services context for these strategies in fragile-state settings, such as Nepal.

**Method:**

A standard situation analysis tool was developed by the PRogramme for Improving Mental health carE (PRIME) consortium to systematically analyze and describe the current gaps in mental health care in Nepal, in order to inform the development of a district level mental health care plan (MHCP). It comprised six sections; general information (e.g. population, socio-economic conditions); mental health policies and plans; mental health treatment coverage; district health services; and community services. Data was obtained from secondary sources, including scientific publications, reports, project documents and hospital records.

**Results:**

Mental health policy exists in Nepal, having been adopted in 1997, but implementation of the policy framework has yet to begin. In common with other LMICs, the budget allocated for mental health is minimal. Mental health services are concentrated in the big cities, with 0.22 psychiatrists and 0.06 psychologists per 100,000 population. The key challenges experienced in developing a district level MHCP included, overburdened health workers, lack of psychotropic medicines in the PHC, lack of mental health supervision in the existing system, and lack of a coordinating body in the Ministry of Health and Population (MoHP). Strategies to overcome these challenges included involvement of MoHP in the process, especially by providing psychotropic medicines and appointing a senior level officer to facilitate project activities, and collaboration with National Health Training Centers (NHTC) in training programs.

**Conclusions:**

This study describes many challenges facing mental health care in Nepal. Most of these challenges are not new, yet this study contributes to our understanding of these difficulties by outlining the national and district level factors that have a direct influence on the development of a district level mental health care plan.

**Electronic supplementary material:**

The online version of this article (doi:10.1186/s13031-014-0030-5) contains supplementary material, which is available to authorized users.

## Introduction

Globally, mental health problems are a serious public health concern accounting for 7.4% of disability adjusted life years (DALY), and 22.9% of all years lived with disability (YLD) [1]. It is estimated that four out of five people with mental illness in Low and Middle Income Countries (LMIC) receive no effective treatment and mental health is often one of the lowest health priorities in those settings [2,3]. Studies have documented several adverse consequences of untreated mental illness, including poverty [4], and premature death [5]. One of the major barriers to scaling up mental health services in LMIC is the scarcity and unequal distribution of specialist mental health professionals [6]. For example, the median number of psychiatrists per 100,000 population in LMIC is 0.05 whereas this number is 8.59 in high-income countries [7]. There is estimated to be a shortage of 1.18 million mental health workers in LMICs alone [6].

In recent years, a number of initiatives have been taken to reduce the treatment gap for mental health problems [8,9]. Evidence is accumulating that mental health services can be delivered effectively by primary health care workers through community-based programs and task-sharing approaches [10,11]. Changing the role of specialist mental health workers (i.e. psychiatrists and psychologists) from a predominant focus on service delivery to also designing and managing mental health services, building clinical capacity of the primary health care (PHC) workers, and providing supervision and quality assurance of mental health services, could help in scaling up mental health services in the LMIC [8,12]. The World Health Organization (WHO) launched the mental health Gap Action Programme (mhGAP) for prioritizing mental, neurological and substance use disorders in 2008 [3]. The aim of mhGAP is to facilitate the delivery of evidence-based interventions by non-specialized health workers in primary health care settings; in addition, mhGAP also advocates scaling up of mental health care through integration of mental health into primary health care [3].

Recently Nepal emerged from a decade-long conflict which claimed the lives of more than 16,000, while many more were subjected to torture, intimidation, extortion, and abduction. Nepal had the highest number of forced “disappearances” in the world in 2003 [13]. The conflict also had an impact on the health system of Nepal. During the period of conflict, health staffs were intimidated and tortured by both the Government forces and insurgents. Vehicles carrying medicine and equipment were stopped so delivery of essential supplies was disrupted [14], which impeded efforts to strengthen the health system and to provide quality health services [15]. Despite the deterioration caused by the conflict, Nepal has made significant progress over the last few years, in terms of improved health status and living standards of the population. The National Health Policy of Nepal, 1991, emphasized the importance of decentralizing the primary health care system to the rural population and providing services in an integrated manner - from the tertiary to sub-health post level – in order to bring about health improvements [16]. Community participation is ensured at all levels of health care through the activities of Female Community Health Volunteers (FCHV). In Nepal’s health care system, Sub Health Posts (SHPs) are the first institutional contact point for basic health services. Altogether, there are 3,129 SHPs in the country which provide essential health care packages and also monitor the activities of FCHVs and other community level health care activities. Health Posts (HP) are the next tier of the health care system and they offer the same package of essential health care services as SHPs, with the additional service of birthing centers. Health Posts also bear responsibility for monitoring the SHPs activities. Altogether there are 676 HPs in the country. SHPs and HPs are not staffed by medical doctors but instead are staffed by Health Assistants (HA), Community Medical Assistants (CMA) and Auxiliary Health Workers (AHW), are responsible for out-patient department (OPD) and emergency management in the HPs and SHPs. In addition, the Auxiliary Nurse Mid-wife (ANM), Village Health Workers (VHW) and Maternal and Child Health Workers (MCHW) are responsible for conducting outreach clinics, immunizations, and maternal and child health, especially antenatal and postnatal care. The third tier of health care comprises the Primary Health Care Centers (PHCC), are an upper-level health care facility established in each electoral area, as a first referral point. The major services delivered within PHCCs are general medical care (including mental health services); family planning, maternal and child health, basic laboratory investigations and provision of the basic health care services that are available in HPs and SHPs. PHCC’s are staffed by medical doctors. The district hospital is the highest level health institution within a district. The District Public Health Office (DPHO) or District Health Office (DHO) is responsible for coordinating health care activities in a particular district area.

Recently, a consortium of research institutions and Ministries of Health in five countries in Asia and Africa (India, Nepal, South Africa, Ethiopia and Uganda), with partners in the UK and the World Health Organization (WHO) established the PRogramme for Improving Mental health carE (PRIME) to study the implementation and scaling up of treatment programs for priority mental disorders in primary and maternal health care contexts [17]. As part of the formative work of PRIME, we conducted a situation analysis to systematically analyze and describe the current gaps in mental health care in Nepal and inform the development of a district level mental health care plan (MHCP). Some of the district level data (PRIME cross country paper) has been published elsewhere [18]; this paper presents in depth data regarding the current mental health situation in Nepal and describes how the national context and district situation are influencing the development of the district level mental health care plan. The results have been interpreted in line with the World Health Organization recommendations for integrating mental health into the primary health care setting [19,20].

## Method

### Setting

Nepal, a landlocked country situated between India and China, is the poorest country in South Asia. The United Nations ranks Nepal 157^th^ out of the world’s 186 countries on the Human Development Index (HDI) with 69.1 years life expectancy at birth [21]. The total population of the country is approximately 28.5 million with the majority (86%) of the total population living in rural areas. Nepal experienced ten years of violent insurgency from 1996 to 2006, initiated by the Communist Party of Nepal Maoist (CPN-M) this was in response to the party’s dissatisfaction regarding gender and caste inequality, and low quality governance. The war formally ended in November 2006 with a comprehensive peace agreement between an alliance of political parties and the Maoists. In 2008, constituent assembly elections were held with the purpose of drafting a new constitution within the following two years. However, after four years, no constitution had been written and consequently the constituent assembly was dissolved in 2012. After a year of political deadlock, a new interim government was formed which successfully completed the election of a second constituent assembly in November, 2013 and handed over power to the newly elected representatives.

### Instrument

A baseline situation analysis was conducted in the five PRIME study sites (Nepal, India, Ethiopia, Uganda and South Africa), to describe the factors relevant to the development and implementation of a district-level mental health care plan [18]. This study used the situation analysis tool developed by the PRIME consortium (http://www.prime.uct.ac.za/images/prime/PRIME_Final_Situational_analysis_Tool.pdf) to collect baseline information relevant to developing a plan to implement and scale up mental health care in Nepal. Details of the development of the situation analysis tool have been reported but the essential components will be outlined in brief [18]. The situation analysis tool emphasizes those factors which were identified by previous reports, as being important for successful integration of mental health into primary health care [22] and those that were required for implementation of the WHO’s mhGAP-IG [23] in the PHC setting. The instrument comprises six sections: Section 1 has 39 items which cover population, economic, health, and social indicators. Section 2 has 15 items including areas such as political support, budget, policy, plan, legislation, benefits and human resources for mental health care. Section 3 focuses on mental health treatment coverage in a district and consists of 19 items related to prevalence of MNS disorders, number of people receiving care and estimated treatment coverage. Section 4, with 62 items, explores district level information about general health services and human resources, mental health care delivered in primary care and health systems structures to support mental health care in PHC. Section 5 has 17 items to document community level information in a district such as; socio-cultural aspects, non-health sector organizations and awareness-raising activities. Section 6 has 4 items related to; health information reporting, monitoring and evaluation systems in a district. The national level data included within the situation analysis tool is initially used to inform the PRIME program being implemented in a single district, and later for discussions regarding the scale-up of services beyond the initial district.

### Process and analysis

The study was conducted between October and December 2011 by the Transcultural Psychosocial Organization (TPO Nepal) (http://www.tponepal.org). We relied mainly on information available in the public domain such as scientific publications, project documents/reports, media reports and hospital records, which was augmented by discussions with district and national level key stakeholders such as government officers, psychiatrists, hospital administration, and other service providers including NGO staff. For example, we reviewed annual reports published by the Department of Health Services (DHO), and District Public Health Office (DPHO), Demographic and Health Survey reports [24], the WHO AIMS report for Nepal [25], and publications from NGOs active in the area. All collected documents and reports explaining the mental health situation or services were reviewed. Information collected from these sources was cross-checked manually to examine the completeness and consistency of the data. Community-level information collected through secondary sources was verified from FCHVs, members of mothers groups, NGO staff and traditional healers. Sources of information, contributing to the situation analysis were documented, together with the date of data collection, allowing the document to be updated when new information was available. When discrepancies between data sources were noted these were cross-checked by the project coordinator, where possible. Information collection from different sources was reviewed and summarized in six broad themes following WHO’s AIMS tool, in order to allow comparison. These themes were policy and infrastructure; mental health services; human resources, mental health in primary health care; public education and links with other sectors; and monitoring and supervision.

## Results

The results of the situation analysis have been presented in Table 1.

**Table 1:** **Mapping of results from situation analysis**

**Category**	**Situation of Nepal**	**Challenges for transformation**
**Policy framework and infrastructure**	• National mental health policy developed in 1997.	• Lack of implementation of the policy framework into practice
	• Policy proposes establishing a separate mental health division in the MoHP	
		• Lack of endorsement of mental health legislation
	• Policy aims to provide mental health services to all the population by 2000	• No mental health division in the MoHP or Department of Health Services (DoHS)
	• Mental health legislation has been drafted	
		• Absence of a long-term mental health strategy and program
	• Mental health is also included under Disability Act and ensures disability benefits to people with severe mental illness	
		• No sufficient budget allocation on mental health
	• Minimal health budget is allocated for mental health	
**Mental health services**	• Mental health services available in the country are mostly institution based	• Lack of holistic treatment approach (community care to specialized care) for the treatment of mental health problems
	• Out of 75 districts, only 7 district hospitals provide mental health services	
		• Lack of integration of mental health services to other non-health sectors such as education, social welfare, and sports
	• Mental health services are limited to psychotropic medication	
		• Lack of separate hospital for providing mental health services to children and elderly people
	• About 450 mental health beds i.e. 1.5 per 100,000 population (both government and private hospitals) in the country	
		• Lack of training and involvement of community volunteers (e.g. FCHVs and traditional healers) in identification and referral of people with mental illness
	• Traditional healers or/and religious leaders are a primary source of mental health treatment in the community	
	• NGOs provide community based mental health services to the specific population; mostly these services are in isolation with government services	
**Mental health services in the PHC**	• No systematic information available about the services of mental health in the PHCs	• Lack of standardized mental health training manual for PHC workers
	• Mental health services are available in few districts where community mental health services are introduced by both government and NGOs	
		• Lack of basic psychotropic medicines in the free drug list
		• Lack of formal referral mechanism from primary to secondary/tertiary care or vice versa
		• Lack of mental health supervision mechanism in the existing supervision system
		• Lack of referral system from primary to tertiary care or vice versa
	• Number of PHC staff trained on mental health by NGOs and government in the country: General Doctors 156 (including doctors from district hospital); Health Assistants/Auxiliary Health Workers (AHWs)/Staff nurse 672; Nurse/auxiliary nurse mid wife 39	
	• Only few (i.e. phenobarbitone and amitryptyline) psychotropic medicines available in the PHCC and HP. No psychotropic medicine available in the SHP	
**Human resource**	• Mental health services are coordinated by different institutions in the center e.g. mental hospital, management division.	• Lack of designated person in the MoHP to coordinate mental health activities
		• Lack of specific strategy for developing human resources in mental health
	• Limited human resource on mental health: 60 psychiatrists 60 (an estimated 25% are reported to be out of the country); 25 psychiatrist nurses; 16 clinical psychologists; 400–500 para-professional counselors (trained by NGOs); 867 general doctors/PHC workers who have received short mental health training	
		• Lack of mental health component in the existing curriculum of all cadres of PHC workers
		• No fulfillment of vacant position in the PHC for long time.
	• Most of the psychiatrists, psychiatrists nurse and psychologists are working in private sector and in big cities	
		• High staff turnover and frequent transfer in another places
	• Community health volunteers are not trained on mental health issue	
**Public education and links with other sectors**	• No mental health education carried out; hence, low/no public awareness on mental health among the general population	• Lack of mass sensitization program on mental health and psychosocial issues in the community
		• Lack of coordination and linkage with other non health sectors such as education and social welfare
	• Mental health highly stigmatized in the community	
	• Psychosocial and mental health services integrated in the hospital based one-stop crisis management center focusing for victims of gender based violence	
		• Lack of involvement of key community actors such as teachers, and youth clubs for identification and referral
	• Psychosocial and mental health incorporated in the care plan of children affected by HIV/AIDS	
	• Mental illnesses is included under disability	
	• Establishment of National Mental Health Network (NMHN) to advocate for mental issues with concerned stakeholders	
	• Establishment of two organizations led by people with mental health and psychosocial disability	
**Monitoring and supervision**	• A morbidity form is available for outpatient clinics, from PHC level	• Lack of involvement of mental health specialists (psychiatrist and psychologist) in the supervision of the PHC workers
	• Current HMIS system includes seven mental health indicators (such as Depression, Psychosis, Anxiety (coded as Neurosis), Mental Retardation, Conversion Disorder (Hysteria), Alcoholism and Epilepsy) in the PHC/district level and 47 mental health problems and 13 self-harm related indicators in hospitals	
		• Lack of nationally representative epidemiological data on mental health
		• Lack of data use culture at the Health Facility level
	• The only national level mental hospital reports comprehensive data on mental health. Besides the above data, it also provides data on bed occupancy rate, mental health budget release and expenditure and leading causes of morbidity.	
	• No separate monitoring and supervision system exists for mental health care	
	• Small scale studies have been conducted with specific population to identify prevalence of mental health problems	

### Policy and legislative framework

A mental health policy for Nepal was developed by the government in 1997 with the commitment of providing basic mental health services to the entire population by integrating mental health care into the existing health care system. The policy framework however, is yet to be implemented; as a result mental health services are essentially limited to a few hospitals, located in the larger cities. The mental health policy proposes several strategies to develop human resources for mental health services and ensure basic human rights of people with mental illness. The mental health legislation which ensures the human rights of people with mental illness was drafted in 2006, but its endorsement by the government is still pending. No clear data were available on the budget amount allocated to mental health and how this money is being spent, because the expenditure occurs in several different ministries including the Ministry of Health and Population and the Ministry of Education. Available data however, indicates that the budget allocated to mental health remains very small, i.e. latest available data reports that less than one percent of the total health budget is spent in mental health [26] and that a substantial proportion of that budget goes to hospital-based services.

### Mental health services

Specialist mental health services were found to be limited to Zonal- or District hospitals. Across the whole country, there are estimated to be around 440 in-patient beds for people with mental illness (combining both governmental, 112 and private hospital facilities, 327); which amounts to 1.5 beds per 100,000 population. No separate in-patient service is available for children with mental illness in Nepal. Due to the geographical circumstances and lack of reliable transportation, Kathmandu (the capital city) or adjoining Indian cities were often the only options available for people with mental illness living in remote districts. In the public sector, counseling or psychotherapeutic services were found to be difficult to access due to the limited number of clinical psychologists. General counseling services are being provided by trained para-professional counselors employed by NGOs; however, such services tend to be limited to specific population groups such as; people affected by conflict, survivors of human trafficking, victims of domestic and gender based violence, and refugees. No systematic data was available regarding the treatment of mental health problems by traditional or religious healers; however, traditional and religious healers are known to be the primary sources of treatment for (mental) health problems in the community [27,28].

### Mental health in primary health care

Mental health services are supposed to be included among the basic health services expected to be delivered in PHC; however, there is limited data indicating delivery of mental health services in the PHC setting in practice. The Management Division, part of the Department of Health Services (DOH), mental hospital and some non-governmental organizations (NGOs) have taken the initiative to train PHC workers in a few districts. The lack of refresher training and non-availability of essential psychotropic medicines, however, has meant that despite the training provided there continues to be a lack of availability of mental health services on a regular basis. Two psychotropic medications, phenobarbitone and amitryptyline, were found to be available in the primary health care centers (PHCC) and, more erratically, at the Health Post (HP) level, but no psychotropic medicine was available at the Sub-Health Post (SHP) level. No counseling or psychotherapeutic services were available through the primary health care system. Due to the lack of clear referral mechanisms from primary to tertiary care, people with mental illness were not being identified and treated effectively, even in the health facilities where there were trained health workers. No standardized training manuals, screening tools or guidelines were available, particularly for the training of PHC workers in detection, diagnosis and treatment of common and severe mental disorders.

### Human resources

The number of mental health specialists was found to be limited and concentrated in large urban areas. An estimated 25% of Nepali psychiatrists (N = 60) are working outside of the country. Data on the number of PHC workers that have received mental health training is incomplete. Available data indicate that around 900 PHC workers, mostly from PHCC level (156 district hospital/ PHC doctors; 672 Health Assistants/Auxiliary Health Workers/Staff Nurse; and 39 ANMs) have received such training. Due to the lack of timely supervision, refresher training and availability of psychotropic medications, most trained PHC workers are not delivering any mental health support services. Female community health volunteers (FCHVs), who are the first contact point of the current public health services, have not received mental health training.

### Public education and link with other services

Mental health problems are highly stigmatized in the community. No mental health awareness programs have been carried out in the public health system. Some NGOs have taken the initiative to run community awareness programs; nevertheless, these programs were targeted to specific populations with limited resources. Due to the lack of a coordinating body in the Ministry of Health, NGO services are implemented in isolation from the public health system. Recently, the Ministry of Health and Population has established ‘one-stop’ crisis centers in some districts of Nepal to provide a comprehensive approach to people suffering from domestic and gender-based violence. Mental health care is a component of this service, but limited to basic psychosocial counseling services. The Ministry of Women, Children and Social Welfare has recently launched a National Minimum Standard (NMS) to ensure comprehensive care and support for survivors of human trafficking and victims of domestic and gender based violence, where psychosocial and mental health services are included as cross-cutting themes [29]. Stigma and discrimination were found to be one of the major barriers for seeking mental health care in the community. Two national-level user organizations have been established in the recent past to advocate for mental health services and the human rights of people with mental illness. A network of most of the organizations working in the mental health and psychosocial fields has also been established for policy advocacy (http://mhnetworknepal.org/).

### Monitoring and research

Mental health indicators have been included in the health management information system (HMIS) from the sub-health post level upwards; although mental health services are not available in either the health post (HP) or sub-health post (SHP). The process of data collection for general health indicators begins at the community level. The Female Community Health Volunteers (FCHVs) use a pictorial register to document health services provided to the community. As these lay health workers are not trained in mental health issues, to date there has not been reporting of any data on mental health. At the PHC and district level either the senior Health Assistant or a medical doctor see the mental health patients and keep records in the out-patient department (OPD) register. Seven different mental disorders, including Depression, Psychosis, Anxiety (coded as Neurosis), Intellectual Disability (coded as Mental Retardation), Conversion Disorder (Hysteria), Alcohol Use Disorder (Alcoholism) and Epilepsy are recorded in the OPD register and reported to the District Health Data Bank located at the District Public Health Office. The data is then reported to a central HMIS section through a HMIS web-portal. Mental health inpatient hospitals and other mental health wards, within the national, regional and zonal hospitals report mental health information to the national HMIS. The annual reports (2008/09, 2009/10 and 2010/11) published by Department of Health Services (DoHS) report data on 47 different mental health problems and 13 items related to self-harm categorized as per ICD-10 definitions. Similar to other health indicators, the quality and accuracy of mental health data collected through HMIS is questioned in regional and national reviews. There are delays in HMIS reporting, sometimes there is under reporting and private health facilities do not regularly report to HMIS.

No national level epidemiological studies have been conducted; therefore no estimates of the national prevalence of mental health problems are available. A few small-scale studies have been conducted mainly by NGOs to identify the prevalence of mental health problems within specific population groups. There is no information regarding the treatment gaps for mental health care.

## Discussion

### Overall situation

This situation analysis demonstrates the scarcity of population-wide mental health services in Nepal. Services are restricted to a few specialists operating mainly in private and/or urban settings. Policies regarding the integration of mental health into primary health care are available; however, these have not been operationalized or implemented. Despite many potential strengths, including the existence of mental health indicators integrated within the national HMIS system, the initiatives of several NGOs to promote community mental health, and the active role of user groups on the advocacy front, the situation of the mental health system is still dire in Nepal. No nationally representative data regarding the prevalence of mental health problems in Nepal are available; however, the studies that have been carried out, show that the prevalence of mental disorders in Nepal does not appear to be different from global prevalence [30-34]. Lack of financial resources and low mental health literacy, in particular misconceptions about mental health problems and stigma associated with mental health problems, contribute to delay or obstruct access to treatment for individuals. In addition, poverty, conflict, displacement and discrimination based on gender and caste/ethnicity, unemployment, and (labor) migration are found to be key risk factors for poor mental health in Nepal [30,35].

### Improvements

Despite this bleak mental health situation, Nepal has made significant improvements in recent years in comparison to a situation analysis conducted by WHO (WHOAIMS) in 2006 [25]. For example; the total number of psychiatrists has increased considerably. The number of mental health beds per 100,000 population was less than 1 (0.8) [25]; now it has been increased to 1.5 per 100,000 population. The involvement of user groups in mental health care, prevention and advocacy have become more established and two national level organizations led by service users have been instituted. Community based mental health programs have been initiated in a few districts by both the government and NGOs [36,37]. In recent years, some initiatives have been taken to include mental health in national health priorities. For example, mental health has been included in the second health sector support program (NHSSP-II); ‘one stop’ crisis centers have been established, and psychosocial and mental health has been included in national minimum standards and standard operating procedures for the care and protection of trafficking survivors [29]. Additionally mental health and psychosocial components in the protection and care for children affected by HIV/AIDS [38]; and the provision of psychotropic medicines in the revised drug lists are key policy level initiatives in mental health and psychosocial care.

### Challenges in developing the district level mental health care plan

In spite of a significant improvement in the situation of mental health care in the last few years; there remain numerous challenges that have impeded the development of the district level mental care plan. Here, we have highlighted key challenges that were identified in the situation analysis and how these challenges were addressed while developing the district mental health plan. First, like other LMIC, there are scarce mental health human resources for providing mental health services in Nepal [39]. Integration of mental health services in the PHC is presented as a strategy to reduce treatment gaps, even though this approach will add more responsibilities to the already overburdened health workers [40]. Rather than providing training to a specific person in the PHC regarding mental health care; we have proposed to train all the PHC workers, to minimize the extra burden of work for one particular person. Secondly, the lack of psychotropic medicines in the PHC has obstructed the availability of mental health services. A memorandum of understanding (MOU) has been signed with the Ministry of Health and Population (MOHP) stating that the essential psychotropic drugs will be supplied through Primary Health Care Revitalization Division (PHC-RD) by allocating the financial budget to the District Public Heath Office (DPHO). Thirdly, a monthly case conference by a psychiatrist from the district hospital has been put in place, to bridge the gap of the current lack of mental health supervision in the existing health care system. Fourthly, considering the current work burden of the PHC workers, the concept of community counselors (i.e. one psychosocial counselor for a certain number of PHCs) has been integrated into the district level MHCP to relieve PHC workers from time-intensive psychotherapeutic services. Fifthly, collaboration with the National Health Training Center (NHTC) has been initiated to conduct training and accredit training certificates for PHC workers, and to ensure that the training manual is in line with government standards. Sixthly, despite the Nepalese government’s commitment to establish a separate division, to coordinate mental health activities as stated in policy, it has not yet been operationalised. The MOHP has agreed to assign a senior level officer in the MOHP to facilitate coordination of the project activities at the national level; this has also been included in the MOU. Seventhly, a Community Informant Detection Tool (CIDT) has been developed to facilitate community detection of mental health problems and increase referral to the PHCs [41]. Female Community Health Volunteers (FCHVs) and mothers group leaders, who are often the first point of contact in their community, have been trained in the use of CIDT. Finally, a referral system has been developed with district hospitals for severe cases in need of specialized care.

### Opportunities for developing district mental health care plan

Globally, several initiatives have taken place to reduce the treatment gap for mental health. Many international and national initiatives and other contextual factors have facilitated the development of district level mental health care plan under PRIME. Firstly, the WHO Mental Health Gap Action Program (mhGAP), has been developed to assist in the scaling-up of mental health services in LMIC [3], and this has been adopted in the Nepalese context to develop the district level mental health care plan. Secondly, the mental health policy (1997) has emphasized the integration of mental health into the PHC by mobilizing existing health workers; and the recent study among the PHC workers has found a positive response and willingness of PHC workers to engage in the process of task shifting [40]. Thirdly, the Nepal health sector support program II (2010–2015) has included mental health components, with the aim of developing a model that could allow integration of mental health into primary health care [42]. Fourthly, initiatives to provide mental health services to specific populations have already been started through the establishment of ‘one stop’ crisis centers in some districts [43]. Fifthly, the Inter Agency Standing Committee (IASC) guidelines on mental health and psychosocial support; which emphasized the value of integrated mental health services, has been translated and pilot-tested in Nepal [44] and Nepal has also signed international conventions including Universal Declaration of Human Rights (UDHR), International Convention of Economic, Social and Cultural Rights (INCESCR) and the Convention on Rights of People with Disabilities (CRPD). Sixthly, a greater need for mental health services in the post-conflict situation has been indicated in a study conducted immediately after the peace agreement between the Government of Nepal and the Maoists which identified a high prevalence of mental health problems in Chitwan and recommended the urgent need for mental health care [30]. Finally, as in other post conflict settings, where post-conflict system restructuring has lead to opportunities for improving mental health care [45,46]; currently several policy developments initiatives (e.g. Health Act, Mental Health Act, Nepal Health Sector Support Program III) are occurring in post conflict Nepal that can be capitalized upon. The current study can contribute to this policy development process, by identifying the areas for improvement, as well as providing some proof of concept of district level mental health care.

### Implications

Recommendations from this situation analysis focus on addressing the identified gaps, particularly for the integration of mental health into the existing health care system. Firstly, it would be easier to coordinate mental health activities and issues with the government, at the central and district level, if a separate mental health unit were to be established in the MoHP. Secondly, considering the risk of overburdening the existing health workers and lack of mental health specialists in many of the districts, we recommend the development of a mid-level mental health and psychosocial cadre within the primary health care system. The concept of mid level mental health professionals has been put into practice in other LMICs such as Uganda [47] and Liberia [48]. Thirdly, as no mental health services are currently available in many of the districts, an efficient referral mechanism should be established to provide specialist mental health services to people with severe mental disorders. Fourthly, non-governmental organizations (NGOs) can play an important role in developing and delivering models for more innovative services; supporting government initiatives and building capacity [49-51]. In Nepal, NGOs have played an important role, especially in providing mental health services for disaster-struck populations, developing community-based mental health care, creating awareness on mental health issues and advocacy [52]. NGOs therefore may need to be involved in the development and implementation of sustainable mental health services. The community at large has a significant role in mental health advocacy and service delivery. Comparisons can be drawn between Nepal and Cambodia as both are war torn countries. The experience in Cambodia of establishing mental health systems highlights the importance of cultural sensitivity and mobilization of local resources, where mental health services were able to be delivered by traditional healers and key community leaders, along with basic mental health care, integrated into primary health care. [53]. Other studies also echo the importance of the involvement of community members as they are culturally sensitive to the context, culture, beliefs and values [54]. Service users and community members may therefore need to be involved in development and implementation of sustainable mental health services.

Our study has some limitations. The most important limitation is that the PRIME situation analysis focuses on readily measurable health system indicators, and data was collected mainly from secondary sources i.e. published data, grey literature, and organization reports. Secondly, this report does not provide an in-depth understanding of attitudinal factors, for example, the acceptability of task-sharing mental health care by health care workers and community members. This issue was addressed in the qualitative formative research, conducted by the PRIME study and has been published elsewhere [40]. Nonetheless, the PRIME situation analysis tool yields data that has value for the planning of integrated mental health services, as well as evaluating the impact of implementing these plans. Further, the core focus of the PRIME situation analysis tool was to collect detailed district level information to map the major gaps to be addressed, by the district mental health care plan. In doing so, only limited national level information was assessed, which is something that should be addressed when using the tool for national level situation analyses.

The strengths of the PRIME situation analyses tool were twofold: (a) it structured the process of development of services to respond to existing gaps, and (b) it engaged local stakeholders by using local data and context as the starting point for the MHCP development.

## Conclusion

This study has underlined many of the known challenges in mental health care in Nepal. However, the results of this study were very helpful for the development of the district level mental health care plan, especially regarding how national context and the district situation can influence the district care plan and what is realistic and feasible given the current lack of capacity at the district level. The key challenges which had influenced the development of the district MHCP included; overburdened health workers, the lack of psychotropic medicines in the PHC especially in the HP and SHP, the lack of mental health supervision in the existing system of care, the lack of a coordinating body in the MoHP, and stigma and discrimination associated with mental illness in the community. Many strategies were adopted to overcome these challenges which included the involvement of MoHP in the program, especially providing psychotropic medicines and appointing a senior level officer to facilitate project activities and collaboration with NHTC. In addition, the lasting effect of the decade-long conflict still exerts an impact on the mental health and psychosocial well-being of people [30,55]. The provision of separate mid level mental health and psychosocial cadre at PHC may need to be considered to meet the level of need. Nepal has seen clear progress in strengthening the mental health care system in the past decade or so, however, major improvements are still required. The weak governance system explains many of the remaining gaps, but also offers opportunities for re-structuring mental health services in post-conflict Nepal.

## Abbreviations

FCHV: Female community health volunteer
HMIS: Health management information system
HP: Health post
LMIC: Low and middle income country
MOH: Ministry of health
MoHP: Ministry of health and population
mhGAP: Mental health gap action program
NGO: Non-Governmental Organization
PHC: Primary health care
PHCC: Primary health care center
PRIME: Programme for improving mental health care
SHP: Sub-health post
WHO: World Health Organization
